# Evaluation of a novel rash scale and a serum proteomic predictor in a randomized phase II trial of sequential or concurrent cetuximab and pemetrexed in previously treated non-small cell lung cancer

**DOI:** 10.1186/1471-2407-14-5

**Published:** 2014-01-04

**Authors:** Michael L Maitland, Matthew R Levine, Mario E Lacouture, Kristen E Wroblewski, Christine H Chung, Ilyssa O Gordon, Livia Szeto, Gail Ratko, Keyoumars Soltani, Mark F Kozloff, Philip C Hoffman, Ravi Salgia, David P Carbone, Theodore G Karrison, Everett E Vokes

**Affiliations:** 1 University of Chicago, Section of Hematology/Oncology, 5841 S Maryland Ave, MC 2115, Chicago, IL 60637, USA; 2 University of Chicago, Section of Dermatology, 5841 S Maryland Ave, MC 5067, Chicago, IL 60637, USA; 3 University of Chicago, Committee on Clinical Pharmacology and Pharmacogenomics, 900 E 57th St, KCBD 6121, Box 11, Chicago, IL 60637, USA; 4 University of Chicago, Comprehensive Cancer Center, 5847 S Maryland Ave, MC 1140, Chicago, IL 60637, USA; 5 Department of Health Studies, University of Chicago, 5841 S Maryland Ave, MC 2007, Chicago, IL 60637, USA; 6 Department of Pathology, University of Chicago, 5841 S Maryland Ave, MC 6101, Chicago, IL, USA; 7 Memorial Sloan-Kettering Cancer Center, 1275 York Ave, New York, NY 10065, USA; 8 Johns Hopkins University, Kimmel Cancer Center, Suite 1100, 401 N Broadway, Baltimore, MD 21287, USA; 9 Vanderbilt University Medical Center, 1211 Medical Center Dr, Nashville, TN 37232, USA; 10 Ingalls Hospital, One Ingalls Dr, Harvey, IL 60426, USA

**Keywords:** Pemetrexed, Lung Cancer, Cetuximab, Rash, EGFR, Proteomics

## Abstract

**Background:**

Candidate predictive biomarkers for epidermal growth factor receptor inhibitors (EGFRi), skin rash and serum proteomic assays, require further qualification to improve EGFRi therapy in non-small cell lung cancer (NSCLC). In a phase II trial that was closed to accrual because of changes in clinical practice we examined the relationships among candidate biomarkers, quantitative changes in tumor size, progression-free and overall survival.

**Methods:**

55 patients with progressive NSCLC after platinum therapy were randomized to receive (Arm A) cetuximab, followed by pemetrexed at progression, or (Arm B) concurrent cetuximab and pemetrexed. All received cetuximab monotherapy for the first 14 days. Pre-treatment serum and weekly rash assessments by standard and EGFRi-induced rash (EIR) scales were collected.

**Results:**

43 patients (20-Arm A, 23-Arm B) completed the 14-day run-in. Median survival was 9.1 months. Arm B had better median overall (Arm B = 10.3 [95% CI 7.5, 16.8]; Arm A = 3.5 [2.8, 11.7] months P = 0.046) and progression-free survival (Arm B = 2.3 [1.6, 3.1]; Arm A = 1.6 [0.9, 1.9] months P = 0.11). The EIR scale distributed ratings among 6 rather than 3 categories but ordinal scale rash severity did not predict outcomes. The serum proteomic classifier and absence of rash after 21 days of cetuximab did.

**Conclusions:**

Absence of rash after 21 days of cetuximab therapy and the serum proteomic classifier, but not ordinal rash severity, were associated with NSCLC outcomes. Although in a small study, these observations were consistent with results from larger retrospective analyses.

**Trial registration:**

Clinicaltrials.gov Identifier NCT00203931

## Background

The monoclonal antibody inhibitor of epidermal growth factor receptor (EGFR)- cetuximab adds modest benefit to front-line chemotherapy in patients with previously untreated advanced non-small cell lung cancer (NSCLC)[1,2]. This benefit is cost-ineffective by reference standards [3,4]. The role of tumor EGFR expression in predicting benefit from adding cetuximab in NSCLC is under investigation [5]–[7]. For the small molecule EGFR inhibitor (EGFRi) erlotinib in NSCLC, skin rash and serum proteomic assays were initially identified as candidate predictive biomarkers [8]–[10]. These findings suggested that non-tumor biomarkers might predict subsets of patients likely to benefit from administration of EGFRis, including cetuximab. A retrospective landmark time-point analysis in the BMS099 trial [2] and a pre-specified subset analysis in the FLEX [7] trial demonstrated better outcomes for patients who developed rash by the end of cycle 1 with cetuximab added to front-line therapy. These findings suggested that rash is a candidate predictive clinical biomarker for cetuximab in front-line NSCLC therapy but to validate the marker would require another large, randomized prospective study [11].

The scales for severity of rash from antineoplastic drugs are crude instruments for determining therapeutic decisions. The scales are structured to equate severity across the full spectrum of adverse events and are designed as tools for safe conduct of clinical trials. They are not validated or intended as biomarkers for treatment effects [12]. To ascertain cetuximab-induced rash, Gatzemeier, et al. collapsed 20 different terms from the MEDRA 10.0 adverse event database [7]. The prospective use of clinician detection of rash as a biomarker might require more structured assessment. The pathophysiology of the rash associated with EGFR inhibitors is most reminiscent of acne rosacea (rosacea)[12], for which a validated severity scale has already been developed [13].

A serum mass spectrometry-based classifier assay segregates candidates for EGFRi therapy into “good” and “bad” prognosis groups [9]. In multiple studies of EGFRis, the patients in the two groups had divergent survival patterns, but not in NSCLC patients treated with cytotoxic therapy, suggesting that the serum proteomic profile is a predictive rather than prognostic marker. In studies of erlotinib in NSCLC: for first-line treatment [14] and when combined with bevacizumab therapy [15] the assay again predicted survival differences. The assay performed similarly in multiple clinical studies of patients with squamous cell carcinoma of the head and neck and in colorectal cancer [16], including subsets of these patients treated with cetuximab. Although commercially available as VeriStrat® (Biodesix, Broomfield, USA), the assay has not been prospectively qualified as a predictive marker for EGFRi therapy.

We conducted a randomized, phase II trial in previously treated NSCLC patients that compared the concurrent and sequential administration of cetuximab and pemetrexed. A component of the trial was to conduct preliminary development of biomarkers for cetuximab. Specifically for this trial, we adapted the validated rosacea severity scale as an EGFRi-induced rash (EIR) scale for better differentiation than the ordinal CTCAE scale of rash assessments. Additionally, serial serum samples were collected and stored at -80°C. The randomization and 2-week run-in design of the trial enabled concurrent analysis of rash rating and the serum assay as candidate predictive markers for cetuximab therapy in NSCLC.

The trial closed prior to achieving its initially intended accrual goals. The original hypothesis to be tested by the trial was to determine in patients with NSCLC refractory to previous chemotherapy whether concomitant treatment with cetuximab and pemetrexed improved progression-free survival compared with cetuximab monotherapy. We estimated a sample of 40 patients per treatment group based on many assumptions, including a relatively modest difference in outcomes between the study arms. Our estimates were inaccurate, and patients who were randomized to the combination therapy arm had much better outcomes than patients in the monotherapy arm. Additionally, large clinical trials of pemetrexed revealed advantages to early initiation of pemetrexed after first-line therapy, and single-arm studies of cetuximab in unselected patients revealed no benefit to cetuximab monotherapy in this population. Therefore the study was closed after reaching half of its intended accrual. The preponderance of available evidence now supports that the administration of pemetrexed in the second-line treatment setting would be superior to cetuximab monotherapy. We report the results of this trial not to demonstrate a definitive assessment of the difference in the randomized treatment arms, but rather to provide the foundation for this biomarker development exercise.

The trial was intended to study pharmacodynamic biomarkers in unselected patients and collection of tumor tissue was not incorporated into the study design. But the study called for all patients to provide pre-treatment serum samples and to undergo standardized, prospective rash evaluations. There are many limitations to the successful development of valid clinically relevant biomarkers concurrently with novel anticancer agents. The need to collect material for markers prospectively, combined with the observation time required to accumulate outcome data with which to qualify biomarkers makes the process slow and expensive. In this manuscript we have examined the data from this trial with 3 goals: 1) to explore the use of quantitative changes in tumor burden at an early time-point in a trial as a measure of drug effect (rather than RECIST-based response or time to progression), 2) to determine whether the prospectively collected biomarker data in this trial are consistent with retrospective analyses of larger clinical trials, and 3) to perform a preliminary estimate of the value of intensive, quantitative rash assessments as a candidate biomarker for future studies of EGFR inhibitors.

## Methods

### Patients

The first patient enrolled in July 2005. The study closed to accrual in March 2008 with minimum 6-month follow-up. Eligible patients were ≥ 18 years with confirmed NSCLC not amenable to curative therapy. All patients were previously treated with at least one platinum- or taxane-containing chemotherapy regimen for locally advanced or metastatic disease. Patients with: prior pemetrexed or prior EGFRi therapy, more than two prior systemic anti-cancer therapies, uncontrolled cardiac disease or pleural/pericardial effusions, and those with the inability to interrupt aspirin or other non-steroidal anti-inflammatory agents for a 5-day period were excluded. Remaining eligibility requirements were as previously described [17]. The study was approved by the institutional review boards of the University of Chicago and Ingalls Hospital and all participants provided written informed consent.

### Study design

#### Treatment

During a 2-week run-in, cetuximab was administered at an initial dose of 400 mg/m^2^ over 120 minutes, followed by weekly infusions at 250 mg/m^2^ over 60 minutes. Typical dose adjustment and discontinuation criteria for cetuximab were used [1,2]. Patients were randomized to treatment assignment at registration. On day 15, subjects assigned to Arm B received 500 mg/m^2^ pemetrexed after cetuximab. Pre-treatment folate and Vitamin B12 supplementation were provided in accordance with the package insert. In case of pemetrexed-related adverse events, doses were initially adjusted to 375 mg/m^2^ and again to 250 mg/m^2^ if required. Pemetrexed every 21 day cycles with weekly cetuximab continued until progression. Patients randomized to Arm A remained on cetuximab monotherapy until progression. Within 1–2 weeks of discontinuing cetuximab, these patients then crossed over to receive pemetrexed and appropriate supportive therapy.

### Assessments

#### Efficacy and safety evaluations

Tumor size was measured by computed tomography (CT) at baseline, on days 34–40, days 55–61, and days 76–82. Thereafter, it was measured every 34–40 days after previous CT scan while patients remained on study. Tumor measurements, responses, and disease progression were assessed using RECIST [18]. The sum of the longest dimensions of each target lesion was tracked throughout the study and used to calculate the change in tumor size as a quantitative assessment of treatment effect [19,20]. Baseline brain MR imaging was not performed. Patients who developed central nervous system symptoms were referred for imaging. Survival was confirmed with treating physicians and the Social Security Index when treating physicians were uncertain of date of death.

#### Rash

Rash assessments were performed at baseline and weekly through the 4th dose of cetuximab, no prophylactic treatment was provided. Rashes were assessed by the same clinician on both the CTCAE version 3 rash/desquamation scale and an EGFRi-Induced Rash Scale (EIR) adapted from the validated rosacea severity scale [13] (Table 1). CTCAE and EIR scales were compared by plotting worst rash rating at any point in the first month of treatment. Uniformly, the highest rating for each patient on each scale occurred at the same time. The maximum change in EIR rating from baseline to day 22 of cetuximab therapy was used to evaluate the relationships among serum proteomic predictor class, changes in tumor size at the first CT imaging evaluation, and rash.

**Table 1:** Rash rating scales used in this investigation

**Grade**	**EGFR-Inhibitor induced rash scale**	**CTCAE Assessment scale**
0	No rash	No rash
1	Isolated pustule or erythematous patch < 1 cm in diameter, on either face/head/neck or chest/back	Macular or papular eruption or erythema without associated symptoms
2	More than one erythematous patch or erythema and telangiectasia on either face/head/neck or chest/back	Macular or papular eruption or erythema with pruritus or other associated symptoms; localized desquamation or other lesions covering < 50% of body surface area
3	More than one pustule or papules on either face/head/neck or chest/back with no associated erythema or telangiectasia	Severe, generalized erythroderma or macular, papular or vesicular eruption; desquamation covering ≥ 50% body surface area
4	Both erythema/telangiectasia and pustules/papules limited to either face/head/neck or chest/back	Generalized exfoliative, ulcerative, or bullous dermatitis
5	Pustules or papules and erythema/telangiectasia present on both the face/head/neck region and the chest/back	Death

#### Serum collection and analysis

Serum samples were collected at baseline and at subsequent time-points but only the baseline samples were analyzed. VeriStrat classifier was determined using coded, de-identified samples without any information regarding treatment assignment as previously described [15]. Codes were returned to the principal investigator with assignments of “good”, “bad”, or “undetermined.” Undetermined samples were treated as missing data in all subsequent analyses.

### Statistical analysis

Progression-free survival (PFS) time was calculated from the date of initial treatment on-study until determination of disease progression radiographically or clinically, or death regardless of attribution except for the analysis looking at the relationship with rash where PFS was calculated from day 22 of study therapy and two patients with progression or death prior to day 22 were excluded. Patients last known to be alive and progression-free were censored at the date of last CT scan without evidence of progression. Based on data available prior to the initiation of the trial, we expected a sample of 40 patients per treatment arm would achieve 80% power at a 0.1 significance level to detect an improvement in the progression-free survival rate at twelve weeks from 50% to 66%.

Progression-free and overall survival times across groups were estimated using the Kaplan-Meier procedure and compared with the log-rank test. In the comparison of overall survival between treatment arms, there was evidence of non-proportional hazards, and therefore the Wilcoxon-Gehan test [21] (which assigns greater weight to earlier time-points) rather than the log-rank test is presented. Results from Wilcoxon-Gehan tests are also reported for the landmark analyses.

### Candidate biomarker evaluation

Given the rapidly changing definitions of standard therapies in NSCLC during the course of the study, it became clear in late 2007 that the enrollment goal would not be achieved, and so the focus of the investigation was reoriented to evaluation of candidate biomarkers in relation to the quantitative assessment of treatment effects with change in tumor size over the first 8 weeks of therapy. For the serum proteomic classifier, 10/43 (23%) evaluable patients did not have a pre-treatment serum marker classification. Fisher’s exact test was used for comparisons of treatment assignment, histology, and sex between those with serum marker data and those without.

During the course of the trial, independent groups conducted modeling studies to determine whether the change in the sum of the longest dimensions of target lesions for NSCLC at 8 weeks of therapy would be an acceptable primary endpoint for phase II clinical trials [19,20,22]. The primary motivations for use of Response Evaluation Criteria in Solid Tumors (RECIST) in phase II clinical trials and the shortcomings of response rate and progression-free survival as endpoints in small randomized trials have been well addressed elsewhere [19,23]–[28]. To maximize our sensitivity for detecting differences in performance of biomarkers for cetuximab, we applied the novel quantitative variable derived from the modeling studies, the log ratio of tumor size at 8 weeks of treatment versus baseline, as an experimental measure of treatment effect in the context of these data. The log of the ratio of the tumor size at the first evaluation on treatment (t1) to the baseline tumor size (t0) was used and defined as follows: log(t1/t0) = log(t1) – log(t0). As previously proposed, non-measurable negative outcomes are assigned poor outcome quantitative values and analyzed using rank-based procedures [19]. Specifically, the one early death prior to the first CT scan on treatment was assumed to have the worst possible outcome, and the four subjects with brain MRIs confirming new metastases at the first evaluation were assumed to be tied with the worst progressor. The nonparametric Wilcoxon rank-sum test was used for comparison of change in tumor size between treatment and serum marker groups. A Spearman rank correlation coefficient was calculated to assess the association between change in tumor size and change in rash.

## Results

### Patient characteristics

Fifty-five patients were enrolled over a three-year period from July 2005 to March 2008. Of these patients, 43 received at least three doses of cetuximab, and were deemed evaluable. The patients are described in Table 2. The drop-out rate was not unusual for a second-line therapy trial in the pre-pemetrexed era, especially because this trial pre-specified that only patients who completed the 3-dose, 2-week run-in of cetuximab would be considered evaluable. Five patients were withdrawn because of infusion reaction to cetuximab, 3 patients withdrew because of inconvenience of commuting to the clinic site, 1 patient could not get pain adequately controlled and withdrew to begin immediate cytotoxic therapy, 1 patient had symptomatic progression of bony metastasis at day 15, 1 patient died suddenly and unexpectedly at week 4, and 1 patient withdrew prior to the first imaging evaluation for intolerability of grade 2 rash. Of the 43 evaluable patients, 33 had pre-treatment samples on which the serum proteomic assessment could be completed. There was no statistically significant difference in missing vs. non-missing patients based on sex (*P* = 0.14), treatment assignment (*P* = 0.29), or histology (*P* = 0.70). There were 21 patients determined to have a “good” serum predictor status and 12 a “bad” status.

**Table 2:** Baseline and disease characteristics for randomized, evaluable patients

**Characteristic**	**Arm A (cetuximab alone)**	**Arm B (cetuximab + pemetrexed)**
	*N* = 20	*N* = 23
**Age (years)**		
Median	62	55
Range	(36–76)	(45–75)
**Sex: No. (%)**		
Men	14 (70)	13 (57)
Women	6 (30)	10 (43)
**Ethnicity: No. (%)**		
White	13 (65)	17 (74)
African-American	7 (35)	5 (22)
Asian	0 (0)	1 (4)
**Smoking history: No. (%)**		
Current	7 (35)	6 (26)
Past	11 (55)	13 (57)
Never	2 (10)	2 (9)
Unknown	0 (0)	2 (9)
**Histology: No. (%)**		
Unspecified	7 (35)	9 (39)
Squamous	6 (30)	8 (35)
Adenocarcinoma	7 (35)	6 (26)

### Clinical outcomes

**Figure 1:**
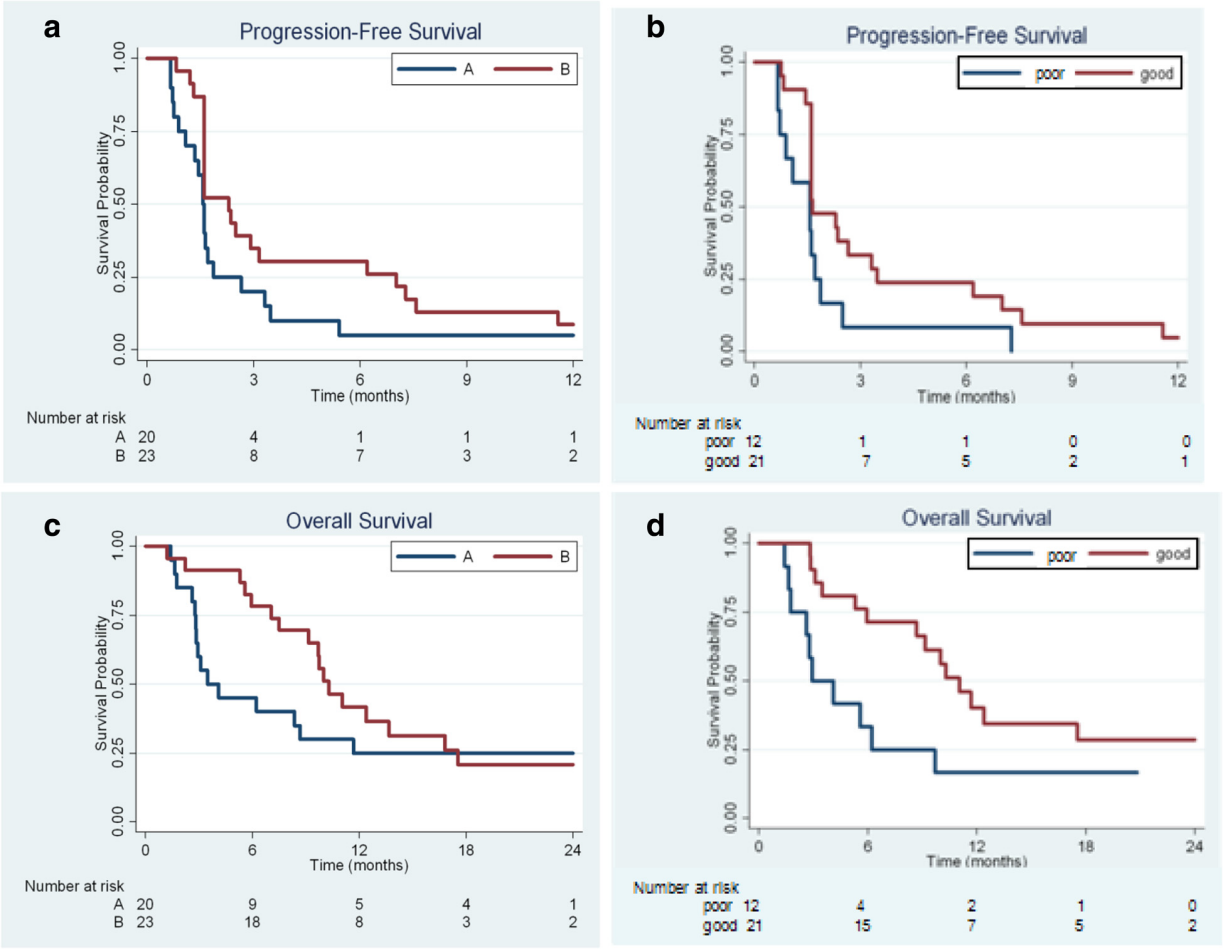
Progression-free and overall survival by study arm (a,c) and by serum assay classifier (b,d).

For the 43 evaluable patients, median PFS was 1.6 months [95% CI 1.6, 2.4] and median overall survival (OS) was 9.1 months [95% CI 5.6, 11.7]. Median PFS for Arm A was 1.6 [95% CI 0.9, 1.9] months, less than the 2.3 months [95% CI 1.6, 3.1] for Arm B (*P* = 0.11) (Figure 1a). Arm B appeared to have superior overall survival (10.3 [95% CI 7.5, 16.8] vs. 3.5 [95% CI 2.8, 11.7] months (*P* = 0.046)) (Figure 1c). Analysis of the effect of the serum proteomic assay classifier suggested superior PFS (*P* = 0.029) (Figure 1b) and overall survival (P = 0.038) (Figure 1d) for the individuals with the “good” classification. Only 25% of those with the “bad” classification were randomized to Arm B while 62% of those with the “good” classification were assigned to Arm B and so in this small study, the treatment arm assignment might confound the assessment of the serum proteomic classifier and vice-versa.

### Rash scales

**Figure 2:**
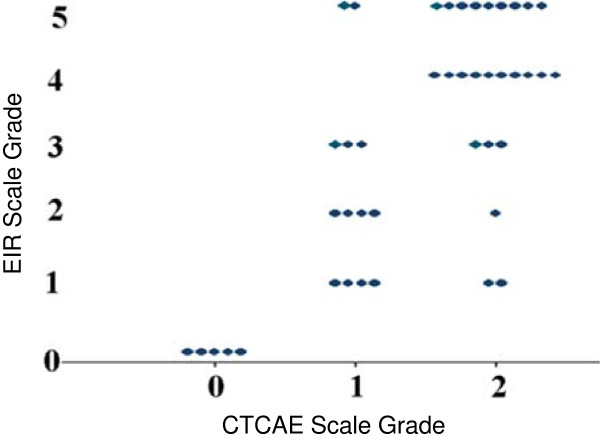
Comparison of worst rash scores per patient by EIR and CTCAE scales.

At each of the pre-specified time-points (pre-treatment, after two doses of cetuximab, after third dose of cetuximab, prior to fourth dose of cetuximab, and every three weeks thereafter) patients were assessed by the same clinician for rash using the two scales; the novel EIR and the CTCAE (Table 1). A comparison of the worst rash rating for each patient by each scale (Figure 2) shows the EIR scale to distribute the typical range of rash more broadly than CTCAE. The CTCAE grade 1 subjects are mostly distributed among 1, 2, and 3 on the EIR scale, while CTCAE grade 2 subjects are distributed primarily among 3, 4, and 5 on the EIR scale.

A landmark analysis was performed to examine the association among PFS, OS and rash development by C1D8 (after 21 days of cetuximab therapy as in Gatzemeier, et al.) with the EIR and CTCAE scales. Using the threshold of rash of grade 1 or higher at any time, ignoring presence of baseline rash, gave the same results on both scales. Rash did not predict improved PFS (*P* = 0.91), but trended toward an association with improved OS (*P* = 0.06).

### Change in tumor size and biomarker evaluations

**Figure 3:**
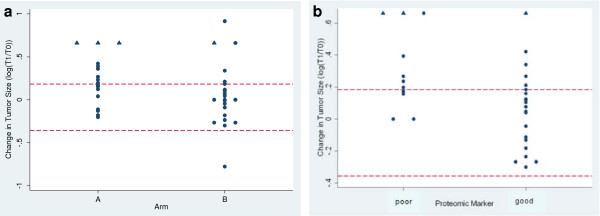
**Change in tumor size by arm (a) and serum proteomic classifier (b).** The log of the ratio of the tumor size at the first evaluation on treatment (T1) to the baseline tumor size (T0) is plotted for each subject. The top red dashed line marks RECIST criteria for progressive disease at the time of the first CT scan, any values at or below the bottom dashed red line meet RECIST criteria for response, and those meeting criteria for stable disease lie in between the two dashed lines. Circles represent actual measured values, while triangle symbols indicate those subjects with brain MRIs confirming new metastases at the first evaluation and therefore equated with the change in tumor size of the worst progressor. The early death in Arm B was assigned a T1/T0 value of 2.5 (log(T1/T0) = 0.92).

Evaluable patients had CT scans conducted pre-treatment and between days 18 to 60; most patients had the initial post-treatment scan performed between days 34–40. We compared the change in tumor size between each patient’s first two scans (Figure 3a) by treatment groups. There was a significant difference in the initial change in tumor size with greater increase in Arm A compared to Arm B (*P* = 0.032), consistent with the findings on PFS between the two groups.

We then tested the associations among this change in tumor size with the serum proteomic classifier and rash. “Good” serum predictor status was associated with more favorable changes in tumor size (*P* = 0.014) (Figure 3b).

**Figure 4:**
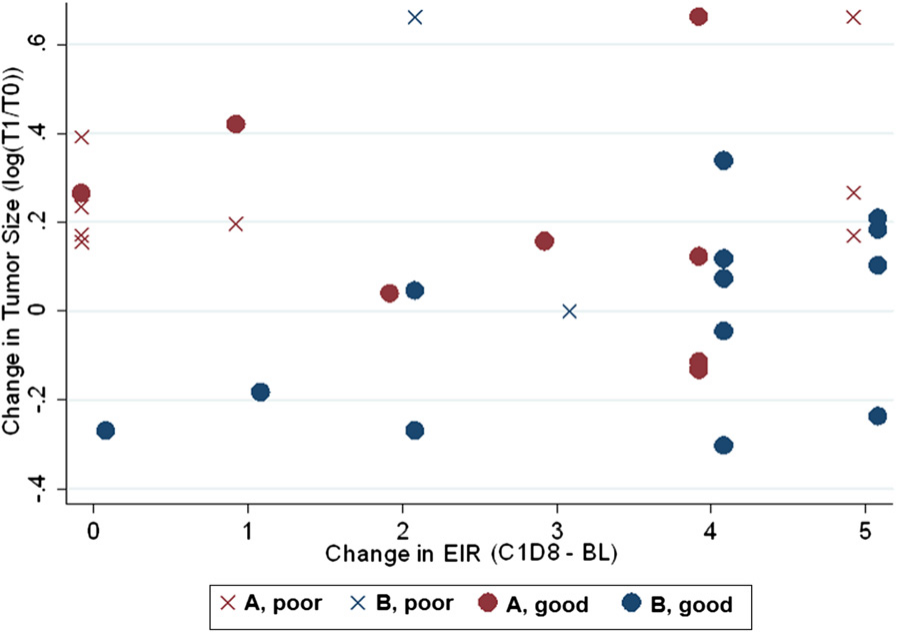
**Change in tumor size vs. change in EIR rash score.** This is a plot of each individual, evaluable patient from this trial. The x-axis represents the change in EIR from baseline to C1D8. The y-axis represents the change in tumor size by log ratio of tumor burden at week 8 to baseline. Each patient is further represented by which arm to which they were randomized (red for Arm A, blue for Arm B) and serum predictor (x for poor, circle for good). The plot reveals potentially informative trends: 1) the top 5 tumor responses (the most negative log ratios) were among subjects randomized to Arm B, all of whom had “good” predictor status, but had rash changes on the EIR of 0, 1, 2, 4, and 5; 2) Of the early progressors (11 subjects with log ratio > 0.18 at or before the first imaging session), 8 had some evidence of rash and 5 had among the most severe rashes with an EIR rating change of 4 or 5; 3) No patient with a “poor” serum proteome predictor had any tumor shrinkage at all, but only 2 of these subjects were randomized to receive pemetrexed at the end of the 2-week run-in.

It was then investigated if a patient’s maximum change in EIR rating from baseline to day 22 of study treatment had any correlation with change in tumor size (Figure 4). The Spearman rank correlation coefficient was 0.15 (p = 0.35, N = 41), indicating no association between rash severity and change in tumor size in this cohort. Given the small sample and the absence of any association between rash and treatment outcome, a test for interaction between rash rating and serum proteomic predictor was not performed. But no individual in the “bad” predictor cohort showed any benefit of cetuximab monotherapy, even those who developed grade 2 through grade 5 rash. The potential value of further distributing rash ratings across a broader ordinal scale is likely to be low.

## Discussion

This exploratory randomized phase II trial with a 2-week run-in of cetuximab monotherapy could inform future development of predictive biomarkers for EGFRi therapy in NSCLC. The biomarker development concepts implemented included: 1) piloting a modified version of the validated rosacea scale for EIR-rating, 2) use of modified quantitative assessment of tumor size changes to assess marker relationships, and 3) concomitant assessment of candidate markers, rash and serum proteomic profiling in the same patients. The EIR scale distributed rash ratings across a larger ordinal range than the conventional CTCAE rash scale; however this scaling did not strengthen the association with treatment outcomes beyond that of the categorical “rash vs. no rash” approach employed by Gatzemeier, et al. [7]. The serum proteomic predictor was associated with treatment outcomes in this study of cetuximab and pemetrexed in the second-line treatment of NSCLC, although this finding might be due to confounding with treatment arm.

As a small trial, not meeting its intended accrual goals, there are clear limitations to this study. The setting of advanced NSCLC meant many patients deteriorated during or immediately after investigational treatment. As the value of information was not clear at the time of conducting the trial, we did not assess candidate molecular markers on patients’ tumors and we did not stratify the randomization by tumor histology. We had competing trials at that time that required information on tissue type for enrollment and a PS of only 0 or 1. This study unintentionally enrolled a typically more ill population with few never smokers (10%), fewer than usual women (under 40%), and many patients with non-adenocarcinoma or unknown histology (70%). The actual sample size meant only large effects, such as the benefit of immediate initiation of pemetrexed, could be detected with conventional measures such as progression-free and overall survival. The randomization did not effectively free the assessment from bias. This is highlighted by the unusually poor median-survival time in Arm A, 3.5 months is even low for a best-supportive care only trial. Arm A had fewer women, had patients with a higher median age, and more patients with serum proteome-profile-predicted “poor” outcomes with cetuximab monotherapy. It is therefore unclear the extent to which the treatment assignment versus the small sample size have biased the outcomes of this trial by study arm. The results are consistent with prior evidence of the value of timely treatment with pemetrexed in second-line treatment of NSCLC [29,30]. Despite not reaching original accrual goals for the study, Arm B subjects, treated concurrently with pemetrexed and cetuximab, had better progression free and overall survival than Arm A subjects treated with sequential cetuximab followed by pemetrexed, reaching statistical significance for the latter. Notably, of the 20 evaluable patients who were randomized to Arm A, 10 did not maintain sufficiently good performance status to receive the pemetrexed after progression.

The EIR scale distributed ratings more broadly than CTCAE, but there is no evidence that this broader distribution will improve upon use of rash severity as a predictive marker for cetuximab efficacy in NSCLC. Serial skin biopsies were performed on a subset of patients in this study, but the heterogeneity of the specimens in terms of estimated volume, estimated total number of cells, enumerated EGFR-expressing cells and ratio of EGFR-expressing to total cells, as well as relative mRNA expression of candidate normalization molecules (data not shown) made semi-quantitative and correlative analyses impossible. Dose-to-rash studies with EGFRi have not revealed a significant benefit for increasing the severity of rash [31,32]. Our data are consistent with the observations of others in larger trials, that incidence and not relative severity of EIR appears to be the pharmacodynamically relevant biomarker [7,10].

Cetuximab monotherapy has no evident value in the treatment in unselected NSCLC patients in the second-line setting. Some could argue that cetuximab therefore has limited if any role in combination therapy. However, the data from Gatzemeier, et al. [7], suggest that a biomarker-based selection of patients who should receive cetuximab added to standard chemotherapy could yield improved outcomes over those reported for the cetuximab arm in the FLEX trial. This circumstance is increasingly common in oncology therapeutics, an agent that has limited but evident benefit in combination cannot be used ethically as monotherapy. Therefore, the early development monotherapy trials become an important opportunity with which to characterize candidate biomarkers and conduct preliminary validation and comparative estimate studies. In this “pure” setting, investigators can determine the typical time course, intensity, and interindividual variance in candidate markers. In a single disease, investigators can conduct preliminary comparisons to make estimates regarding which markers represent the best opportunities for future validation and qualification studies in large trials, including combination therapy trials.

For future qualification of candidate markers of EGFRi in the treatment of NSCLC, we propose that either the serum proteomic assay or incidence of rash be further evaluated as a means to exclude patients from receipt of cetuximab therapy. In patients who have the “poor” proteomic profile and those who fail to develop rash by 21 days of cetuximab therapy the likelihood of benefitting from cetuximab therapy appears low. In NSCLC, these markers, similar to the use of *KRAS* mutations in colorectal cancer, have reproducibly associated with absence of benefit from EGFRi therapy [14]–[16,33]. Our findings suggest future strategies to qualify these biomarkers for clinical use would be to demonstrate prospectively in a randomized trial that either or both markers effectively reduces the unnecessary, toxic, ineffective, and expensive use of cetuximab [11]. Ideally, this study should help to identify safe and more effective alternatives for the patients who will not benefit from cetuximab therapy.

## Conclusions

Typical phase II trials of combination therapy have had limited impact on the overall development of cancer therapeutics [34]. Here we have demonstrated a strategy of: a brief monotherapy run-in, randomization, concurrent assessment of candidate biomarkers, and implementation of quantitative tumor size assessments as a potential means to make a local phase II trial more informative. The results of this study suggest that future development of either EIR or a serum proteomic predictor assay might focus on qualifying these markers to exclude prior to or early in treatment patients who have a low likelihood of benefiting from these expensive, potentially toxic therapies.

## Competing interests

CHC participated in an ad hoc advisory board meeting for and received compensation from Biodesix during the conduct of this investigation.

## Authors’ contributions

MLM conceived of the initial protocol design with EEV and together drafted the protocol. In the first year of the study EEV served as principal investigator and MLM served the remaining years, coordinated efforts of the co-authors on sample and statistical analyses and interpretation and with MRL organized data, drafted all figures, and the first draft of the manuscript. MEL conceived and developed the EIR rating scale, contributed to the design and conduct of the trial and performed serial skin biopsies on initial patients enrolled in the study and KS assumed those responsibilities for the remainder of the study. KEW performed statistical analyses and drafted part of the manuscript. CHC supervised all analyses with the serum proteomic predictor, interpreted study results and modified the manuscript. IOG supervised analyses of skin biopsies and participated in interpretation of rash rating results. LS and GR provided patient care and ensured adherence to the study protocol. MFK, PCH, and RS enrolled patients, provided patient care, performed rash ratings and disease response assessments. DPC contributed to study design, provided funding and technical support on serum proteomic predictor analyses. TGK developed the initial study design and supervised KEW in all study-related analyses and interpretations. All authors read, commented upon and approved the final manuscript.

## Pre-publication history

The pre-publication history for this paper can be accessed here:

http://www.biomedcentral.com/1471-2407/14/5/prepub
